# The Role of Long Noncoding RNAs in Central Nervous System and Neurodegenerative Diseases

**DOI:** 10.3389/fnbeh.2018.00175

**Published:** 2018-09-28

**Authors:** Chang-Wei Wei, Ting Luo, Shan-Shan Zou, An-Shi Wu

**Affiliations:** Department of Anesthesiology, Beijing Chao-Yang Hospital, Capital Medical University, Beijing, China

**Keywords:** long noncoding RNAs, central nervous system, neurodegenerative diseases, regulatory mechanism, gene expression

## Abstract

Long noncoding RNAs (lncRNAs) refer to a group of noncoding RNAs (ncRNAs) that has a transcript of more than 200 nucleotides in length in eukaryotic cells. The lncRNAs regulate gene expression at epigenetic, transcriptional, and post-transcriptional levels by multiple action modes. In this review, we describe the diverse roles reported for lncRNAs, and discuss how they could mechanistically be involved in the development of central nervous system (CNS) and neurodegenerative diseases. Further studies on the function of lncRNAs and their mechanism will help deepen our understanding of the development, function, and diseases of the CNS, and provide new ideas for the design and development of some therapeutic drugs.

## Introduction

Over the past decade, the extensive applications of second-generation sequencing technology have led to the discovery of tens of thousands of RNA transcripts that have similar properties to mRNAs, but are not translated into proteins. Long noncoding RNA (lncRNA) is a kind of noncoding RNA (ncRNA), with a length of longer than 200 nucleotides, that lacks a significant open reading frame (ORF) encoding a protein (Sun and Kraus, 2015). The central nervous system (CNS) is the most highly evolved and sophisticated biological system. The development of the CNS is a complex arrangement of stem cells, growth/differentiation factors, transcription factors, and epigenetic control. It consists of a large number of neuronal and glial cell subtypes distributed in rigorous and precise regions, forming a dynamic neural network that responds to internal signals and external stimuli and then is responsible for mediating the complex functional repertoire of the CNS including performing higher level functions, for example, cognition and behavior (Graff and Mansuy, 2008). As one of the most abundant ncRNA classes, lncRNAs are derived from different locations in the genome for transcription and are highly expressed in the brain (Mercer et al., 2008; Qureshi et al., 2010). The role of lncRNAs has been validated in brain development, neuronal function, maintenance, and differentiation.

Neurodegenerative diseases are associated with multiple clinical manifestations, brain pathologies, and health consequences (Quan et al., 2017). They include relatively well-known conditions like Alzheimer's disease (AD), Parkinson's disease (PD), and Huntington's disease (HD). In particular, AD and PD are a group of typically late-onset, progressive disorders that lead to cognitive and/or movement disorders (Peden and Ironside, 2012). Although drug therapy and/or surgery can delay the progression of these diseases, most neurodegenerative diseases remain untreatable. In addition, neurodegenerative diseases represent an increasing financial burden on health care systems, which attempt to respond to an aging population. Therefore, there is an urgent need to develop methods for preventing or curing neurodegenerative diseases. Some efforts carried out by the scientific community revealed important insights into the molecular bases of these disease, but the specific mechanism remains unknown. Increasing evidence has suggested that lncRNAs are involved in the pathogenesis of neurodegenerative diseases (Johnson, 2012; Briggs et al., 2015; Riva et al., 2016). This review summarizes data on lncRNA expression in the central nervous system (CNS) and neurodegenerative diseases and focuses on the role of some specific lncRNAs, which may provide new insights into our understanding of the etiology and pathophysiology of the neurodegenerative diseases.

## Biology of lncRNAs

### Definition of lncRNAs

Genomewide analysis of the eukaryotic transcriptome revealed that up to 90% of the human genome IS transcribed. However, the GENCODE-annotated exon of the protein-encoding gene covers only 2.94% of the genome, while the rest are ncRNAs (Djebali et al., 2012). Noncoding transcripts are further divided into house-keeping ncRNAs and regulatory ncRNAs. House-keeping ncRNAs include ribosomes, metastasis, small nuclei, and small nucleolar RNA. Regulatory ncRNAs are generally divided into two classes based on nucleotide length. Those <200 nucleotides are commonly referred to as short/small ncRNAs, including microRNAs (miRNAs), small interfering RNAs, and Piwi-related RNAs, and those >200 nucleotides are known as lncRNAs (Nagano and Fraser, 2011).

The lncRNA transcripts are partially similar to messenger RNAs (mRNAs) as they are frequently transcribed by RNA polymerase II, contain classical splice sites (GU/AG), have an mRNA-like structure that contains intron and exon structures, exhibit alternative splicing, no open reading frame (ORF) in the sequence, and are associated with the same types of histone modifications as protein-coding genes (PCGs)(Ponting et al., 2009). They also have a specific secondary structure that provides multiple sites for protein binding or the specific binding between DNA and RNA by the principle of base pair complementarity. The main sources of lncRNAs are from PCG-related regions (Magistri et al., 2012), gene regulatory regions (Hung et al., 2011; Mercer et al., 2011), and specific chromosomal regions (Azzalin et al., 2007). The lncRNA was originally thought to be the “noise” of genomic transcription and as not having biological functions. However, recent studies have shown that lncRNAs can regulate gene expression at epigenetic, transcriptional, and post-transcriptional levels, and participate in X-chromosome silencing, genome imprinting, and chromatin modification, transcriptional activation, and many other important biological processes (Singh and Prasanth, 2013; Goff and Rinn, 2015; Kazemzadeh et al., 2015). Until now, only very few lncRNAs have been validated by experiment, while most of the lncRNAs have been annotated via bioinformatics and still need further experimental verification.

### Expression and sequence conservation of lncRNAs

In 2002, Japanese scientists revealed that in large-scale sequencing of a mouse full-length complementary DNA (cDNA) library, a large number of ncRNA transcripts were identified (Okazaki et al., 2002). However, due to lack of functional annotation, these RNA transcripts did not attract the attention of researchers during subsequent periods. Not until 2007 did the situation change. Rinn et al. in Stanford University reported a 2.2-Kb functional lncRNA gene (Hox transcript antisense intergenic RNA, *HOTAIR*) (Rinn et al., 2007). It was found that *HOTAIR* could interact with the protein complex polycomb, which can modify chromatin, inhibit the transcription of the *Hox* gene, and regulate the growth and development of organisms. In 2008, Mercer et al. used *in situ* hybridization to identify the expression of a large amount of lncRNAs in mouse brain (Mercer et al., 2008). The expression levels of these lncRNAs are associated with specific neuroanatomical locations, cell types, and subcellular locations. For example, *Evf2* is mainly expressed in the ventral forebrain. The tissue-specific expression of lncRNAs also includes *Hox* transcript antisense intergenic RNA myeloid1 (*HOTAIRM1*), which is specifically expressed in the bone marrow (Zhang et al., 2009) and *Msx1* antisense RNA (*Msx1-AS* RNA), which is expressed only in differentiated teeth and bone cells (Coudert et al., 2005). The expression level of *Msx1-AS* RNA is negatively correlated with the content of Msx1 protein (Babajko et al., 2009).

The lncRNAs can appear in different subcellular structures, and the proportion of those lncRNAs located in the nucleus is the largest. For example, lncRNA *MEN* ε*/*β is mainly located in the nucleus, and is an important component of nuclear substructure paraspeckles (Sasaki et al., 2009; Sunwoo et al., 2009). Furthermore, metastasis-associated lung adenocarcinoma transcript 1 (*MALAT-1*) and nuclear-enriched abundant transcript 1 (*Neat1*) are localized mainly in the nuclear speckle of the nucleus, and are associated with the cleavage of RNA precursors (Hutchinson et al., 2007; Tripathi et al., 2010). In addition, Cesana et al. reported that *linc-MD1* is mainly expressed in the cytoplasm of differentiated muscle cells, and regulates the differentiation of skeletal muscles as a competing endogenous RNA (ceRNA) (Cesana et al., 2011). In 2011, Rackham et al. first identified three lncRNAs (*lncND5, lncND6*, and *lncCytb*) encoded by mitochondrial genome DNA in the analysis of high-throughput sequencing data (Rackham et al., 2011). This shows that lncRNAs may exist in many subcellular structures. Hence, special subcellular localization plays an important role in the biological function of lncRNAs.

The sequence conservation of lncRNAs is low. The sequence similarity is close to the intron region of the PCG, lower than 70% in humans and mice, and slightly lower than that in the 5′UTR or 3′UTR of genes (Pang et al., 2006). It was revealed in some studies that low sequence conservation did not affect the functional conservation of lncRNAs. Illustrated by the examples of *Xist* and *HOTAIR* expressed in mammals, although they are not highly conservative in sequence, the roles in X chromosome dosage compensation and epigenetic silencing are the same (Braidotti et al., 2004; Pauler et al., 2005). In addition, some lncRNAs with high sequence homology have also been found such as *MALAT1* and *Neat1* (Nakagawa et al., 2012; Zhang et al., 2012).

## The lncRNAs in CNS development

The CNS development is a complex and stereotyped process that requires the precise spatiotemporal regulation of pluripotent stem cell proliferation and differentiation. Neurons are able to change their set of synaptic connections and the relative strength of each of these connections over time in response to sensory experience and other environmental cues. The dynamic expression of lncRNAs plays an important role in controlling these processes (Table 1).

**Table 1:** Molecular mechanisms of lncRNA in neuronal differentiation and synaptogenesis.

**lncRNAs**	**Biological function**	**Molecular mechanisms**	**References**
*RMST*	Promotes neuronal differentiation	The RMST interacts with *SOX2* to regulate neurogenic genes including *ASCL1* and *DLX1*	Ng et al., 2013; Lee et al., 2017
*Linc-Brn1b*	Specifies cortical NSPC fate and regulates area patterning and layer formation of mouse neocortex	Deletion of the linc-*Brn1b* locus leads to significant decrease in Brn1 expression	Sauvageau et al., 2013; Wang et al., 2017
*Tuna*	Regulates pluripotency and neural differentiation of ESCs	*TUNA* formed a complex with three pluripotency-related RNA-binding proteins, PTBP1, hnRNP-K, and NCL	Lin et al., 2014
*Gomafu*	Controls retinal development; Dysregulated in schizophrenia	*Gomafu/Miat* regulates splicing of neuronal genes, including *DISC1, ERRB4*, and *WNT7B*, probably via association with splicing factors SF1, SRSF1, and QKI	Briggs et al., 2015; Wang et al., 2017
*KCNA2-AS*	Regulates neuronal firing properties	It dynamically downregulates expression of the *KCNA2* potassium channel subunit	Zhao et al., 2013
*BDNF-AS*	Depletion of BDNF-AS promotes neuronal outgrowth adult neurogenesis; BDNF-AS modulates synaptic function	*BDNF-AS* negatively regulates BDNF expression by recruiting EZH2 (a PRC2 core component) in response to neuronal depolarization	Modarresi et al., 2012
*Malat1*	Promotes dendrite maturation and synaptogenesis in cultured hippocampal neurons	*Malat1* associates with SR family splicing factors to control expression of synaptic molecules including Nlgn1 and SynCAM1	Wang et al., 2017
*BC1/BC200*	Regulates synaptic excitability	Controls the translational repression of specific target mRNAs within synapses through a mechanism involving direct recruitment of translational machineries	Zhong et al., 2009

*RMST, rhabdomyosarcoma 2 associated transcript; ASCL1, achaete-scute family bHLH transcription factor 1; DLX1, distal-less homeobox 1; Tuna, TCL1 upstream neural differentiation-associated RNA; PTBP1, polypyrimidine tract binding protein 1; hnRNP-K, heterogeneous nuclear ribonucleoprotein K; NCL, nucleolin; DISC1, disrupted in schizophrenia 1; EZH2, enhancer of zeste 2 polycomb repressive complex 2 subunit; PRC2, proteasome component 2*.

### Molecular mechanisms of lncRNAs in cell proliferation and differentiation

A study has revealed that approximately 40% of lncRNAs are distributed in the CNS (equivalent to 4,000–20,000 lncRNA genes) (Briggs et al., 2015). It may be that brain complexity requires more regulatory RNA to maintain normal brain development and function. The lncRNAs are involved in the regulation of the proliferation and development of the nervous system, and enable the nervous system to proliferate and differentiate according to normal time and spatial orders (Amaral et al., 2013).

The lncRNAs are involved in the differentiation of embryonic cells into neural cells during the embryonic period (Klattenhoff et al., 2013). A genetic analysis of the embryo revealed that lncRNAs are closely associated with the coding genes involved in neuronal differentiation and cell morphological maintenance, such as brain-derived neurotrophic factor (*BDNF*), developing brain homeobox 1 (*DBX1*), neuron–glia-related cell-adhesion molecule (*Nrcam*), etc. (Lv et al., 2013). Studies on neural stem cells revealed that lncRNAs are involved in regulating the differentiation of stem cells into neural cells (Ng et al., 2012; Antoniou et al., 2014; Ramos et al., 2016). For example, Ng *et al*. revealed that *lncRNA-ES1, lncRNA-ES2*, and *lncRNA-ES* are associated with the maintenance and differentiation of neural stem cells. Guttman et al. discovered at least 1,000 conserved intergenic lncRNAs genes by analyzing the chromatin of mouse neurons (including neural precursor cells) in 2009; they also primarily hypothesized and verified the role of lncRNAs in the maintenance of embryonic stem cells (Guttman et al., 2009). Subsequently, functional genomic analysis revealed that these intergenic lncRNA genes were not only involved the differentiation of mouse ventral forebrain-derived neural stem cells, but also in brain aging, mouse hippocampal development, differentiation of gamma aminobutyric acid (GABA)-ergic neurons, oligodendrocyte myelination, and the calcineurin-dependent signaling pathway, by regulating the expression of some important genes (Mercer et al., 2010).

A recent study has revealed that lncRNAs are associated with PCGs in neural development and play an important role in maintaining the intrinsic morphology and characteristics of neurons (Roberts et al., 2014). For example, the sex-determining region Y-box 2 (*Sox2*) is an important regulator of neural stem cell differentiation and nerve growth. *Sox2OT* encodes a sense-orientation transcript that overlaps with the *Sox2*. The genomic proximity of *Sox2OT* and *Sox2* suggested a possible regulatory role for *Sox2OT* in the neural stem cell differentiation and regeneration of neural cells.

### The lncRNAs and synaptic plasticity

Synaptic plasticity is the basis of learning and memory and plays a key role in maintaining the stability of the nerve pathway. Some studies found that many lncRNAs might be involved in the regulation of synaptic plasticity (Leal et al., 2014; Panja and Bramham, 2014; Maag et al., 2015). Angelman syndrome (AS) is caused by the deletion of or an inactivating mutation in the maternal E3 ubiquitin ligase (*UBE3A*) gene and is characterized by intellectual disability, severe developmental delays, and speech impairment (Mabb et al., 2011). Furthermore, due to the expression of lncRNA *UBE3A-ATS*, the paternal allele of *UBE3A* undergoes silencing. A study found that hippocampal long-term potentiation (LTP) is defective in the AS-mutant mouse model (Jiang et al., 1998). Similar plasticity deficits may be the cause of learning disabilities observed in AS patients. This important role of *UBE3A-ATS* in neuronal function highlights the possible role of lncRNA in memory formation, while further studies on the role of other lncRNAs in these processes are necessary. The normal development of gamma-aminobutyric acid-ergic (GABAergic) inhibitory interneurons in the hippocampus is the key to learning in embryonic and adult brains. The *Evf-2* lncRNA, which transcribed from the Dlx-5/6 ultraconserved region, is essential for GABAergic neuron development. The *Evf-2* acts through the Dlx-2 transcriptional coactivator to increase the transcriptional activity of Dlx-5/6 and glutamate decarboxylase 1 (Gad1 required for the conversion of glutamate to GABA) (Colasante et al., 2008), and then regulates the gene expression of GABAergic interneurons in the developing mouse brain. The *Evf-2* silencing leads to abnormal synaptic activity in mice through abnormal formation of GABAergic circuits in the hippocampus and dentate gyrus (Bond et al., 2009).

In neurons, local protein synthesis in synaptodendritic microdomains has been implicated in the growth and plasticity of synapses. The lncRNA brain cytoplasmic 200 (*BC200*) is selectively located in the dendrites of postsynaptic neurons, and regulates local protein synthesis by blocking transcription initiation, thereby, controlling signal transduction (Kondrashov et al., 2005). In addition, *BC1* is an lncRNA located in the dendrites of neurons, which is a specific repressor of translation. Experimental use of internal ribosome entry mechanisms and sucrose density gradient centrifugation showed that BC1-mediated repression targets translation at the level of initiation. Specifically, *BC1* RNA inhibited formation of the 48S preinitiation complex, i.e., recruitment of the small ribosomal subunit to the mRNA (Wang et al., 2002). After that, the authors demonstrated that the lack of *BC1* could induce nerve overexcitation (Wang et al., 2005).

Neurons can change their connections when they face changes in the environment. Alterations in components of ion channels or signal proteins may affect neuronal excitability or neuronal function. For example, the magnitude of the action potential can be affected by changing the subunit stoichiometry of potassium channels. Potassium voltage-gated channel subfamily A member 2 (*KCNA2*) is a major potassium channel subunit, and its expression is regulated by overlapping antisense RNA when facing peripheral nerve injury or neuralgia. In a healthy rat model, *KCNA2* antisense (*KCNA2-AS*) is expressed in few dorsal root ganglia (DRG) neurons (<20% of DRG), and *KCNA2* is expressed in most DRG neurons. However, in the face of peripheral nerve injury, a large amount of *KCNA2-AS* is induced by transcription factor myeloid zinc finger 1 in DRG neurons. *In vivo* and *in vitro* experiments have revealed that the overexpression of *KCNA2-AS* could decrease KCNA2 mRNA and protein content. The mechanism may be that it binds with competing DNA- or RNA-binding factors, thereby, regulating the expression of *KCNA2* (Zhao et al., 2013). This regulation alters the function of DRG neurons. Therefore, *KCNA2-AS* can respond to peripheral nerve damage by altering synaptic plasticity. The lncRNA *NEAT1* provides a scaffolding function in the nucleus that releases regulatory proteins after neuronal activation to finetune excitatory responses and correlate with pathological seizure states. In addition, downregulation of lncRNA *NEAT1* results in changes in the expression of multiple gene transcripts involved in ion channel function following neuronal activation (Barry et al., 2017).

The BDNF is a class of secreted growth factors that are essential for neuronal growth, synaptic plasticity, and participation in learning and memory processes (Leal et al., 2014; Ninan, 2014; Zagrebelsky and Korte, 2014). The BDNF is an important growth factor, not only being regulated by miRNA, but also regulated by lncRNA. Dissection of the human BDNF locus revealed that antisense transcription of the *BDNF* gene from anti-BDNF (*BDNF-AS*, also annotated as *BDNF-OS*) in the brain takes place to form dsRNA duplexes with BDNF mRNA. Inhibition of *BDNF-AS* by antagoNAT *in vivo* or siRNA *in vitro* both resulted in increased *BDNF* mRNA and protein levels, which promoted neurite outgrowth and maturation, suggesting that anti-BDNF plays an important role in *BDNF* function (Lipovich et al., 2012; Modarresi et al., 2012). Further studies revealed that *BDNF-AS* inhibits *BDNF* transcription by recruiting the zeste homolog 2 (EZH2) and polycomb suppression complex 2 (PRC2) enhancers to the *BDNF* promoter region (Pruunsild et al., 2007).

The lncRNA *Gomafu* is widely expressed in the brain, (Mercer et al., 2008) and has recently been shown to modulate alternative splicing of the schizophrenia-associated genes *DISC1* and *ERBB4* (Barry et al., 2014). Since deletion of *Erbb4* in mice enhances LTP in the hippocampus, it is important to determine whether *Gomafu*-regulated splice isoforms affect normal synaptic plasticity (Pitcher et al., 2008; Shamir et al., 2012). In addition, lncRNA *MALAT1* mediates spinal cord maturation and synapse formation by recruiting splicing factors. In cultured hippocampal neurons, knock-down of Malat1 reduces synaptic density, whereas overexpression leads to autonomous increase in cells at synaptic density (Bernard et al., 2010).

These findings suggest that lncRNAs can regulate the synaptic plasticity, and thus, the fidelity of cognitive and memory processes by dynamically monitoring and integrating multiple transcriptional and post-transcriptional events.

## The role of lncRNAs in neurodegenerative diseases

The lncRNAs have broad-spectrum functions in the normal brain development and function maintenance. It is not surprising that dysregulation of lncRNAs might play a pivotal role in neurodegenerative diseases. This view has been reinforced by the identification of a growing number of lncRNAs that directly regulate the expression of genes associated with neurodegenerative disorders, including AD, PD, HD, respectively.

### The correlation of lncRNAs and alzheimer's disease

The AD is a neurodegenerative disease with cognitive decline as the main clinical manifestation. Its pathological mechanism has not been completely understood to date. The main pathological characteristic of AD is the progressive disease of neurons accompanied by the loss of neurons, senile plaques (SPs), and neurofibrillary tangles (Dewachter et al., 2000; Ghosal et al., 2009). Senile plaques mainly consist of β-amyloid (Aβ). Amyloid precursor protein was abnormally cleaved by β-amyloid precursor protein cleaving enzyme 1 (BACE1) to produce Aβ. The excessive accumulation of Aβ produces neurotoxicity. The *BACE1-AS* is an lncRNA transcribed from the antisense strand of the *BACE1* gene, which is highly expressed in the brain of patients with AD. A study revealed that the expression of *BACE1-AS* was significantly increased under extracellular stimulation, such as Aβ_42_ (Faghihi et al., 2008). However, *BACE1-AS* does not inhibit the transcription of mRNA by forming dimers through binding with the coding genes as general natural antisense transcripts (NATs). In contrast, *BACE1-AS* covers the binding site of *miR-485-5p* on *BACE1* mRNA, thereby, silencing the inhibition of *miR-485-5p* on BACE1 mRNA and increasing the stability of *BACE1* mRNA (Faghihi et al., 2010). This leads to the production of more Aβ_42_ and an increase in the formation of SPs in the brain in AD patients, aggravating the development of the disease. Feng et al. found that the level of the *BACE1* is increased in the plasma of AD patients and that it has a high specificity (88%) for AD, indicating *BACE1* may be a potential candidate biomarker to predict AD (Feng et al., 2018). Recently, Yang et al., investigated the hippocampal expression patterns of dysregulated lncRNAs in a rat model of AD through microarray (Yang et al., 2017). The authors identified a total of 315 lncRNAs and 311 mRNAs significantly dysregulated in the AD model, such as *BC158567, MRAK050857, Mrak033976*, etc. These differentially expressed genes are involved in synaptic transmission regulation, cholinergic regulation, and CNS neuron differentiation, all of which are important in learning and memory, as well as the development of AD. Furthermore, the dysregulated lncRNAs in the AD group are involved in the neuroactive ligand–receptor interaction, the renin–angiotensin system, axon guidance, and the PI3K–Akt, MAPK, and mTOR signaling pathways. Among these, the PI3K–Akt, MAPK, and mTOR signaling pathways play important roles in long-term learning and memory.

The BC200 RNA is selectively located in neuronal synapses, which regulates the synthesis of proteins surrounding the postsynaptic membrane. Mus et al. revealed that the expression of *BC200* RNA was significantly downregulated in the prefrontal cortical area in normal elderly subjects (49–86 years old, by autopsy), and was significantly increased in the brain of AD patients. Further studies have revealed that the distribution of *BC200* RNA in the brain of AD patients has changed. The *BC200* RNA in the brain of AD patients is located in the perinuclear area in the cluster, instead of the synaptic terminals. Hence, it loses its regulatory function on proteins surrounding the postsynaptic membrane. The overexpression and error spatial localization of *BC200* RNA may excessively inhibit the synthesis of cytoplasmic proteins, thereby, aggravating the pathological changes of AD (Mus et al., 2007).

The *17A* is also an lncRNA that has been recently discovered. It is located in intron 3 of the G protein-coupled receptor-51 (*GRP51*) gene, regulates the production of *GRP51* variable transcript, and inhibits the canonical transcription of GAGA(B2) receptors, thereby, significantly affecting the GABA-B signaling pathway. The inflammatory reaction in the brain in AD patients can activate the expression of *17A*. This increases the secretion of Aβ and the Aβ42/Aβ40 ratio, aggravating the progress of the disease (Massone et al., 2011). Another lncRNA *51A* overlapping with *SORL1* (antisense) was also shown to affect Aβ formation and upregulated in AD (Ciarlo et al., 2013).

In addition, neurotrophic factors (NTFs) play an important role in maintaining nervous system function. The expression level of *BDNF* is significantly changed in neurodegenerative diseases, psychosis, and neurodevelopmental disorders (Lu et al., 2013, 2014; Song et al., 2015). The inhibition of *BDNF-AS* expression can increase the expression of BDNF in the brain, which has a wide prospect for the treatment of neurodegenerative diseases. Glial cell-derived neurotrophic factor opposite strand (*GDNFOS*) is transcribed from the antisense strand of the GDNF gene. A study revealed that there was a difference in the expression of GDNFOS subunit in AD brains compared with normal brains, but the underlying mechanisms remain unknown at present (Airavaara et al., 2011). Furthermore, another study found that *Sox2OT* and *1810014B01Rik* could serve as biomarkers in the early and late stages of neurodegenerative diseases (Arisi et al., 2011). Tremendous efforts have been put into their translational applications by identifying specific lncRNAs that are changed in Alzheimer's disease in order to provide biomarkers and better illustrate molecular pathways.

### The correlation between lncRNAs and parkinson's disease

Parkinson's disease (PD) is a neurodegenerative disease that commonly occurs in the elderly. Its pathological characteristic is the degeneration of dopaminergic neurons in the substantia nigra-striatum system, which decreases dopamine secretion, resulting in a series of extrapyramidal responses. The maintenance of mitochondrial homeostasis plays an important role in the progression of PD (Moreira et al., 2010; Jin et al., 2014; Luo et al., 2015). Gene studies revealed that PD family-related genes such as α*-synuclein, parkin*, PTEN-induced putative kinase 1 (*PINK1*), *DJ-1*, and leucine-rich repeat kinase 2 (*LRRK2*) were closely related to mitochondrial function (Puspita et al., 2017). Endogenous *PINK1* is localized in the mitochondrial membrane and plays an important role in energy metabolism in neurons and muscle cells. In addition, *PINK1* can also inhibit the release of cytochrome C from mitochondria and decrease the occurrence of apoptosis. The inhibition or overexpression of *PINK1* can lead to abnormal mitochondrial morphology and affect the release of dopamine, resulting in behavior defects (Petit et al., 2005). Study found that lncRNA *NEAT1* was significantly upregulated in the midbrain of PD mice, and that lncRNA *NEAT1* promoted MPTP-induced autophagy in PD by stabilizing PINK1 protein (Yan et al., 2018). Noncoding antisense PTEN-induced putative kinase 1 (*naPINK1*) is an lncRNA transcribed from the antisense strand of the *PINK1* gene, which can stabilize the expression of the *PINK1* variable transcript *svPINK1*. The silencing of *naPINK1* leads to the decrease in *svPINK1* in neurons. This suggests that the PD process can be improved by regulating the *PINK1* locus (Scheele et al., 2007).

Ubiquitin carboxy-terminal hydrolase L1 (*Uchl1)* is a neuron-restricted protein acting as a de-ubiquitinating enzyme or a monoubiquitin stabilizer. The *UCHL1* gene mutations have been discovered to be related to familial PD and the oxidative inactivation of Uchl1 protein has been reported in PD and AD brains (Choi et al., 2004). The *Uchl1-AS* is a nuclear-enriched lncRNA that is transcribed antisense to the mouse *Uchl1*. The *Uchl1-AS* increases the protein synthesis of UCHL1 at the post-transcriptional level and then regulates the progression of PD (Carrieri et al., 2015). Furthermore, the activity of *Uchl1-AS* is controlled by the signaling pathway. The *Uchl1* mRNA is mainly localized in the cytoplasm, while *Uchl1-AS* is abundant in the nucleus of dopaminergic neurons. Interestingly, the mTOR inhibitor-Rapamycin treatment resulted in the induction of Uchl1 protein by association of shuttling *Uchl1-AS* from the nucleus to the cytoplasm, suggesting that the interaction between Uchl1–ncRNA–mTOR may be critical for PD development (Carrieri et al., 2012; Vucićević et al., 2014).

### Correlation between lncRNAs and huntington's disease

Huntington's disease (HD) is a rare autosomal dominant inherited neurodegenerative disease. Its pathological change is the loss of neurons in the striatum and cortex in brain. There is a CAG repetitive sequence in exon 1 of its pathogenic gene huntingtin (*HTT*), which encodes polyglutamine. The excessive repeat amplification of CAG in the gene-coding region induces the prolongation of the polyglutamine chain in protein, thereby inducing lesions (Aziz et al., 2011; De Souza and Leavitt, 2015). The *HTT* can regulate the nuclear translocation of transcription inhibiting factor RE-1 silencing transcription factor (REST), which is also known as neuron-restrictive silencer factor (NRSF)(Shimojo, 2008). Mutation in HTT leads to the abnormal nuclear/interstitial translocation of REST/NRSF, thereby, inducing the abnormal expression of the downstream target gene of REST/NRSF, including PCGs and ncRNA. Through studies on the expression profile of brain tissue in HD patients, Johnson et al. revealed that the expression of *HAR1* lncRNA in striatum decreased significantly, and the main cause was that REST inhibited *HAR1* transcription by locating in the *HAR1* locus *via* the specific DNA regulatory element (Johnson et al., 2010). Chung et al. discovered an lncRNA-*HTTAS* transcribed from the antisense strand of *HTT*. This has two types of transcripts: *HTTAS-v1* (exons 1 and 3) and *HTTAS-v2* (exons 2 and 3), where exon 1 contains repeated gene loci. Cell level verification results revealed that the overexpression of *HTTAS-v1* could significantly decrease the transcription level of *HTT*, while *HTT* transcription level significantly increased after *HTTAS-v1* was disrupted by siRNA. Furthermore, *HTTAS-v1* expression was found to be downregulated in frontal cortex of HD patients, and this strongly suggests that the change in *HTTAS* may play a certain role in the progress of HD (Chung et al., 2011).

The lncRNA *TUG1* has been shown to be a direct downstream target of p53, which is known to be upregulated in HD itself. Therefore, *TUG1* appears to be a pro-survival factor in neurons. The upregulation we observed in HD - probably through p53 activation–actually acts against mutHTT cytotoxicity (Khalil et al., 2009). The *NEAT1* is a nuclear-enrich lncRNA that is essential for the formation and maintenance of paraspeckles, which are subnuclear bodies found in mammalian cells (Clemson et al., 2009). Sunwoo et al. found that the levels of *NEAT1* were increased in R6/2 mice and HD patients. In order to determine the biological effects of *NEAT1* on neuronal survival, the authors transfected neuro2A cells with the *NEAT1* short isotype vector and subjected them to H2O2-induced damage. The *NEAT1* transfected cells showed enhanced viability under oxidative stress, confirming that upregulation of *NEAT1* contributes to neuroprotective mechanisms against neuronal damage rather than pathology of neurodegenerative diseases (Sunwoo et al., 2017). It has been reported that *MEG3* is a REST target and is dynamically expressed during neurodevelopment and associated with PRC2 chromatin regulators. This supporting *MEG3* might be participating in chromatin regulation, noncoding transcription, and neurodevelopment in HD (Johnson, 2012). Although functional studies on *DGCR5* have not been performed, the fact that this neuro-specific disease-related transcript is directly targeted by REST suggests that it has important functions in the human nervous system (Sutherland et al., 1996; Johnson, 2012).

### Summary of lncRNAs in neurodegenerative diseases

An increasing number of studies report on lncRNAs as being implicated in neurodegenerative diseases, including AD, PD, and HD (Table 2).

**Table 2:** Dysregulated lncRNAs in neurodegenerative diseases.

**lncRNAs**	**Direction of change**	**Relative disease**	**Biological function**	**References**
*Sox2OT*	Up	AD, PD	Regulate cotranscribed *Sox2* gene expression to down neurogenesis.	Arisi et al., 2011; Shimozaki, 2014
*BC200*	up(Soma) down(Dentritic)	AD, PD	Modulate local proteins in postsynaptic dendritic micro-domains to maintenance of long-term synaptic plasticity.	Lukiw et al., 1992; Mus et al., 2007; Vucićević et al., 2014; Luo and Chen, 2016
*BACE1-AS*	up	AD	Increase *BACE1* mRNA stability resulting in additional Aβ42 generation through a post-transcriptional feed-forward mechanism.	Faghihi et al., 2008; Liu et al., 2014
*NAT-Rad18*	up	AD	Down the expression of DNA repair protein Rad18 resulting in the neuron being more sensitive to apoptosis.	Parenti et al., 2007
*NDM29*	up	AD	Promotes Aβ secretion with increased Aβ42/ Aβ40 ratio, expression induced by inflammatory processes.	Massone et al., 2012
*51A*	up	AD	Promotes the alternative splicing of *SORL1* and increases Aβ formation.	Ciarlo et al., 2013; Tan et al., 2013; Luo and Chen, 2016
*17A*	up	AD	Impair GABAB signaling pathway by decreasing *GABAB R2* transcription.	Massone et al., 2011
*GDNFOS*	Dysregulated/differences in tissue expression patterns	AD	Modulate the expression of endogenous *GDNF* in human brain.	Airavaara et al., 2011
*naPINK1*	up	PD	Stabilize the *svPINK1*, resulting in disturbed mitochondrial respiratory chain, and then increase the sensitivity to apoptosis.	Chiba et al., 2009; Sai et al., 2012
*PINK1-AS*	-	PD	Positively regulates the stability of *svPINK1* transcript.	Scheele et al., 2007
*UCHL1-AS*	-	PD	Positively regulates *UCHL1* mRNA translation.	Carrieri et al., 2012, 2015
*HAR1*	down	HD	Necessary for retinal development, the function with HD not be mentioned.	Johnson et al., 2010; Johnson, 2012
*HTT-AS*	down	HD	*HTTAS-v1* specifically reduces endogenous *HTT* transcript levels.	Chung et al., 2011
*MEG3*	down	HD	Binds the PCR2 epigenetic silencing complex.	Johnson, 2012

*AD, Alzheimer's disease; PD, Parkinson's disease; HD, Huntington's disease; Sox2OT, Sox2 overlapping transcript; BACE1-AS, β-site amyloid precursor protein cleaving enzyme 1 antisense; GDNFOS, glial cell derived neurotrophic factor opposite strand; HAR1, human accelerated region 1; HTT-AS, Huntingtin antisense; PINK1-AS, PTEN induced putative kinase 1 antisense; UCHL1-AS, ubiquitin C-terminal hydrolase L1 antisense; MEG3, maternally expressed 3; NDM29, Neuroblastoma Differentiation Marker 29*.

The roles of *BACE1-AS* lncRNA have been widely defined in AD. The *BACE1-AS* levels are upregulated in AD brains, where *BACE1-AS* acts by stabilizing BACE1 mRNA, thereby, increasing BACE1 protein content and Aβ42 formation. While lncRNA *BC200, 17A, BC158567, MRAK050857, Mrak033976, BDNF-AS, Sox2OT*, and *1810014B01Rik* are also involved in AD. In PD, the lncRNA *UCHL1-AS1* acts by directly promoting translation of UCHL1 protein leading to perturbation of the ubiquitin–proteasome system. Different lncRNAs, such as *naPINK1, NEAT1 PINK1-AS, BC200*, and *Sox2OT*, were found to be dysregulated in their expression also in PD. Several studies reported altered expression levels of known lncRNAs *HTT-AS, TUG1, NEAT1, MEG3*, and *DGCR5* in the brains of HD patients. The emerging role of lncRNAs in neurodegenerative diseases suggests that their dysregulation may trigger neuronal death through an unexplored RNA-based regulatory mechanism that needs to be further investigated.

## Challenges and perspectives

With the continuous deepening of gene regulation research and the emergence of more biological methods, lncRNA function and mechanism will be further elucidated.

This relatively poorly characterized class of RNAs, with little or no coding capacity, has been implicated in the growth and development of the nervous system, the differentiation of neurons, synaptic plasticity, and the occurrence and progress of many diseases. Although many studies have been performed in clinical patients using various disease models, the exact role and impact of lncRNAs in disease pathogenesis still remain obscure. At present, there are still challenges for our understanding of lncRNAs.

Our lack of understanding of the molecular mechanisms of lncRNA action makes it particularly difficult for us to recognize the biological function of lncRNA. As we move forward from the annotation of lncRNAs to more emphasis on molecular function and biology, we still do not fully understand the biological significance of lncRNAs as a group. We need better tools to track lncRNA localization across the genome, monitor lncRNA interactions with proteins and nucleic acids, and determine the structure and elucidate the key structure–function relationships of lncRNAs, especially how they interact with proteins. Furthermore, unlike PCGs with systemic functional annotation systems, the lack of an annotation system for lncRNA function makes it difficult to evaluate computational algorithms for functional prediction. Finally, is it useful to find lncRNAs in neurodegenerative diseases? Two major long-term challenges in disease research are (a) the development of noninvasive diagnostic methods for monitoring the progression of neurodegenerative diseases, and (b) the development of treatments to cure and reverse disease processes.

In fact, more and more attention has been paid to lncRNAs as potential targets for disease biomarkers or therapeutic strategies. Indeed, several commercial entities targeting lncRNAs (such as OPKO-CURNA and RaNA therapeutic agents) have been developed to design and develop oligonucleotide therapeutics for the treatment of neurodegenerative diseases (Qureshi and Mehler, 2013). Hereby, further investigation into the role of lncRNAs will provide a better understanding of how the brain functions and how diseases develop, and lead to greater insights into further therapeutic development for neurodegenerative diseases based on manipulations of lncRNA functions.

## Author contributions

C-WW substantial contributions to the conception and design of the work, the acquisition, analysis, interpretation of data for the work. C-WW drafting the work and revising it critically for important intellectual content. A-SW final approval of the version to be published. TL and S-SZ agreement to be accountable for all aspects of the work in ensuring that questions related to the accuracy and integrity of any part of the work are appropriately investigated and resolved.

### Conflict of interest statement

The authors declare that the research was conducted in the absence of any commercial or financial relationships that could be construed as a potential conflict of interest.
